# Effectiveness of digital technologies to engage and support the wellbeing of people with dementia and family carers at home and in care homes: A scoping review

**DOI:** 10.1177/14713012231178445

**Published:** 2023-05-26

**Authors:** Lyndsey Bradley, Shanti Shanker, Jane Murphy, Lee-Ann Fenge, Michelle Heward

**Affiliations:** Ageing and Dementia Research Centre, Faculty of Health & Social Sciences, 276175 Bournemouth University, Bournemouth, UK; Centre for Seldom Heard Voices, Faculty of Health & Social Sciences, 276175 Bournemouth University, Bournemouth, UK; Ageing and Dementia Research Centre, Faculty of Health & Social Sciences, 276175 Bournemouth University, Bournemouth, UK

**Keywords:** wellbeing, engagement, coproduction, digital technologies, home, care home, person centred, outcome measure, social relationships, connection

## Abstract

Use of digital technologies to support meaningful engagement of people with dementia and carers increased during the COVID-19 pandemic. The purpose of this scoping review was to determine the effectiveness of digital technologies in supporting the engagement and wellbeing of people with dementia and family carers at home and in care homes. Studies published in peer reviewed literature were identified across four databases (CINAHL, Medline, PUBMED, PsychINFO). Sixteen studies met the inclusion criteria. Findings indicate that digital technologies can potentially support the wellbeing of people with dementia and family carers, although only a few studies had measured impact on wellbeing, as many were reporting on technology at proof-of-concept stage rather than commercially ready products. Moreover, current studies lacked meaningful involvement of people with dementia, family carers, and care professionals in the design of the technology. Future research should bring together people with dementia, family carers, care professionals and designers to coproduce digital technologies with researchers and evaluate them using robust methodologies. Codesign should start early in the intervention development phase and continue until implementation. There is a need for real world applications that nurture social relationships by focusing on how digital technologies can support more personalised, adaptive forms of care. Developing the evidence base to identify what makes digital technologies effective in supporting the wellbeing of people with dementia is crucial. Future interventions should therefore consider the needs and preferences of people with dementia, their families, and professional carers, as well as the suitability and sensitivity of wellbeing outcome measures.

## Introduction

Digital technologies have a fundamental role across support systems in dementia care (Block et al., 2020; Ekström et al., 2017). In the United Kingdom (UK), take up of digital technologies by people with dementia and their carers increased during the COVID-19 pandemic (Chricico et al., 2022). During the pandemic, UK Government restrictions to avoid the spread of the coronavirus disease forced many to stop socialising and into long periods of isolation and loneliness. Digital technologies proved a ‘lifeline’ of support as they enabled individuals to access critical support and engage in communication, which minimised the impact caused by the social restrictions (Chirico et al., 2021; Goodman-Casanova et al., 2020). This demonstrated the potential of digital technologies in enhancing wellbeing and reducing mental health risk factors (Chirico et al., 2021; Cuffaro et al., 2020). For people with dementia, digital technologies played an integral role in upholding their selfhood and wellbeing by maintaining connections with family and professional carers (Goodman-Casanova et al., 2020; Ludden et al., 2019). Using digital technologies to support the wellbeing of people with dementia living at home and in care homes, is an important step in transforming future care delivery. Utilisation of digital technologies for wellbeing should begin as early as possible to ensure people are familiar and comfortable with such technologies. Early adoption may also ameliorate the transition from home to care homes, or other residential settings, and so our focus in this paper is on both home and care homes.

### Digital technologies that support meaningful engagement

Meaningful engagement aligns with person-centred care and highlights the importance of the ‘doing’ of a diverse range of activities, as well as the subjective meaning placed upon those activities by the person (Du Toit et al., 2018; Hammell, 2004; Neal et al., 2020). In this review, we focus on digital technologies used by or with people with dementia, which we refer to as ‘active’ digital technologies. These can be contrasted with the more ‘passive’ digital technologies designed to monitor people and their environment, such as digital movement sensors and blood pressure monitors. Active digital technologies support the meaningful engagement of people with dementia and caregivers and include digital reminiscence, life story books, social networking, and robotics. The evidence of the effectiveness of existing interventions is emerging. A recent review identified twenty studies focused on the use of robotics (n = 14) and multi-media computer programs (n = 6) for enhancing meaningful engagement of people with dementia living in care home environments (Neal et al., 2020). The authors noted a lack of consistency across studies in how activity, interaction, and engagement were measured, which lead to conflicting results in relation to the positive impact on meaningful engagement (Neal et al., 2020). Further work is currently underway to identify the barriers and facilitators to implementing digital technologies that support meaningful engagement of people with dementia in care home environments (Luscombe et al., 2021). Whilst another review determines whether using technology can reduce loneliness in people with dementia living at home and in care homes. Findings suggest that most studies (n = 64/73) used technology to replace face-to-face interactions with other people in the same setting, rather than providing an opportunity to connect people in different locations (Anderson et al., 2022).

Moreover, there are several methodological issues within current studies with not all studies involving people with dementia and family carers in the development of digital technologies, which has implications for acceptability, usability, longevity, and effectiveness (Sanders and Stappers, 2008). Previous reviews advocate involving people with dementia and family carers throughout the entire development process as equal partners or co-designers, rather than just in the evaluation phase which has been typical in many studies (Holthe et al., 2018; Luscombe et al., 2021; Meiland et al., 2017; Span et al., 2013; Topo, 2009). In addition, there is a lack of consistency in outcome measures used to determine engagement. To ascertain the validity of such criticisms, we describe the outcome measures used and explore the level of involvement of people with dementia and family carers in the studies that we have included in this review.

### Defining wellbeing for people with dementia

Enabling people with dementia to ‘live well’ is a global policy and research priority (Clarke et al., 2020). Yet, the term wellbeing has different cultural meanings across the world, and rarely has there been a consensus for this term. Operationalising a definition of wellbeing has been problematic for people with neurodegenerative diseases such as dementia, due to assumptions that health is a key part of wellbeing, for example, Cambridge University Press (2019) describes wellbeing as ‘*… the state of feeling healthy and happy’*. Aiming to separate wellbeing from health, a recent paper reviewed the term ‘wellbeing’, proposing a global definition: ‘*Wellbeing is a state of positive feelings and meeting full potential in the world. It can be measured subjectively and objectively using a salutogenic (i.e., a sense of coherence supporting coping,*Mittlemark et al., 2022; Harrop, et al., 2006; Sarabia-Cobo & Sarriá, 2021) approach (Simons and Baldwin, 2021: p990). The authors argue that adding prefixes such as physical, mental, and social is unnecessary as it creates a further overlap between health and wellbeing (Simons and Baldwin, 2021). Conversely, other scholars have recognised that psychological, social, and emotional well-being are three overlapping and overarching domains for the measurement of wellbeing in people with dementia (Clarke et al., 2020). Rather than measuring outcomes of psychosocial interventions from a symptom-focused, loss/deficit approach, or from the broader quality of life concepts, Clarke et al., (2020) provide an alternative asset/strengths-based conceptual framework of wellbeing in dementia (Clarke et al., 2020). They argue that equilibrium and a potential state of ‘flourishing’ can be measured despite the challenges of dementia and ageing related multi-morbidities (Clarke et al., 2020). We explore this further by defining the wellbeing emphasis (social, emotional, physical, and psychological) for the studies included in this review.

### Aim of review

The aim of this review is to examine digital technologies literature in response to the following research question: *To what extent are digital technologies effective in engaging and supporting the wellbeing* o*f people with dementia and family carers at home and in care homes?*

## Methods

### Nature of review

Guided by the Preferred Reporting Items for Systematic Reviews and Meta-Analysis (PRISMA), a scoping review was conducted to identify the effectiveness of digital technology for supporting the wellbeing of people with dementia and their carers (Tricco et al., 2018). This method of scoping review was selected as it lends itself to address broader research questions by systematically searching and retrieving data to synthesise knowledge and highlight gaps in the literature (Arksey & O’Malley, 2005).

### Inclusion criteria

Article inclusion was based on a broad set criterion linked to our research question. This helped to ensure all relevant literature were incorporated. The SPIDER strategy (Sample, Phenomenon of Interest, Design, Evaluation, Research type) tool which is an adapted version of the PICO tool (Population, Intervention, Comparison, Outcome) (Cooke et al., 2012), guided our search as follows:

• Sample: people with dementia and family carers
• Phenomenon of Interest: active digital technologies-based interventions in own home or care home
• Design: case study, observational study, randomized controlled trial, quasi-experimental study, questionnaire, interviews, and focus groups.
• Evaluation: outcomes related to the mental, physical, social and/or emotional well-being of the person with dementia.
• Research type: quantitative, qualitative, or mixed method.

Empirical studies were included but study protocols, conference papers, published thesis and review articles were excluded. We excluded articles not in English language and without access to the full paper. Due to the rapidly changing nature of digital technology, we focused on articles published between 2015 and 2021.

### Literature search

A comprehensive search was conducted by one author (LB), with support from two co-authors (MH, SS) and the University librarian in June 2021. Four databases were searched (CINAHL, Medline, PUBMED, PsychINFO), and the search strategy incorporated Boolean Operators and truncation along with including medical terminology via the MESH and explode function in Medline to search keywords: (Digital Tech* or Technology) AND (Dementia or Alzheimer’s Disease) (Supplementary Material). These searches were merged with the inclusion criteria keywords including (care* or patient or person or health professional) AND (care* or community or home).

### Data extraction and synthesis

Articles were assessed accordingly based on meeting the inclusion criteria by three authors (LB, SS, MH). All papers were initially screened based on the title and abstract. The remaining articles were subjected to a full text review. Where there was disagreement on whether to include an article, the remaining authors (JM, LF) were consulted to inform the decision.

Data relating to the included studies were extracted and charted including design, demographics, care setting, data collection, methods, measures, and findings. The quality of included studies has not been assessed as the nature of this review was to map current evidence and identify gaps. The papers highlighted different approaches, methods and digital technologies being used. Therefore, results are presented through a narrative synthesis, with findings from the studies summarised as follows (i) characteristics of included studies (ii) type of interventions (iii) involvement of people with dementia and carers.

## Results

**Figure 1.**
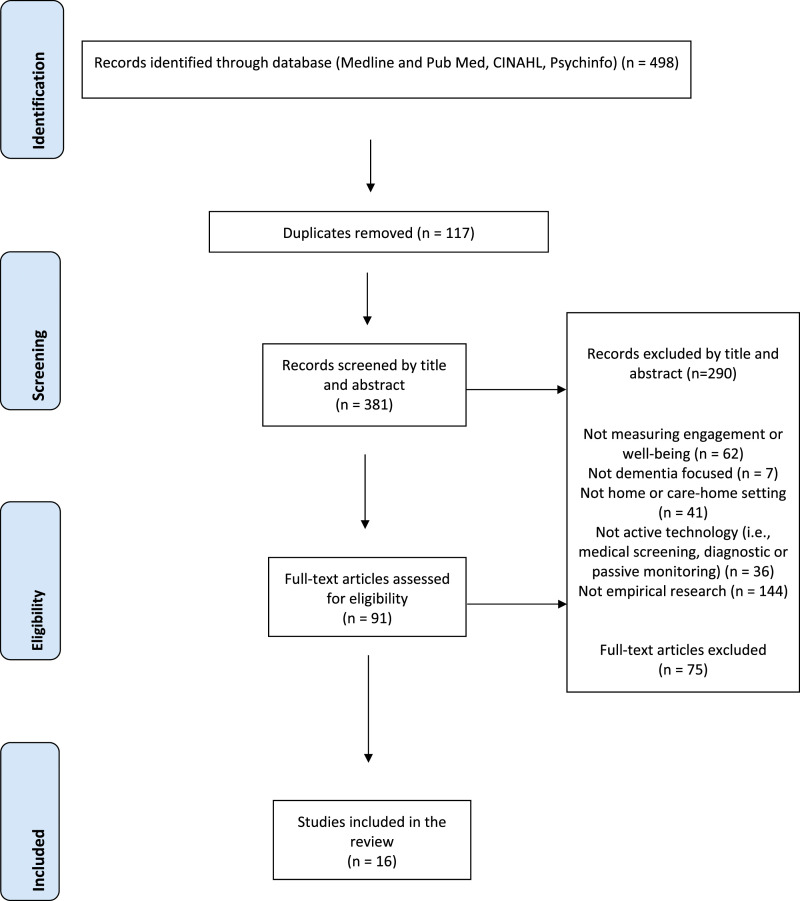
PRISMA flow diagram summarizes study identification and selection.

A database search returned 498 articles, with 381 articles remaining after duplicates removed. Of those, 290 articles were excluded after screening titles and abstracts. Of the remaining 91 articles selected for full-text review, we identified 16 articles eligible for inclusion. The PRISMA flow diagram summarizes study identification and selection (Figure 1).

### Characteristics of included studies

The key study characteristics are outlined in Table 1. Studies were undertaken in Australia (n = 4), the Netherlands (n = 3), the United States of America (n = 2), Canada (n = 1), Italy (n = 1), Mexico (n = 1), Norway (n = 1), Norway and Portugal (n = 1), New Zealand (n = 1), and the United Kingdom (n = 1). Study setting included own home (n = 6) and care home (n = 10). Intervention and study designs were heterogeneous as discussed below. Three studies were part of Randomised Control Trials (RCTs) (Jones et al., 2018; Jøranson et al, 2016; Moyle et al, 2017). Five studies were based solely on quantitative methods, including three observation studies (Chu et al., 2017; Feng et al., 2020; Lancioni, et al., 2016) and two with pre and post outcome measures (Bemelmans et al., 2015; Laird et al, 2018). Five studies were based solely on qualitative methods, including two observational studies (Begum et al., 2015; Cruz-Sandoval and Favela., 2019) and three interview based studies (Darragh et al., 2017; Goodall et al., 2021; Wang et al., 2017). Three studies drew on mixed quantitative and qualitative methods: one using quantitative (outcome measures) and qualitative (interviews) (Lazar et al, 2016) and two drawing on quantitative (pre-test – post-test survey and qualitative (interviews) (D'Cunha et al., 2021; Wilkerson, et al., 2018). In terms of sample size, the largest study involved 415 participants and the smallest was 10. Three studies reported on samples of more than 100 participants (Chu et al., 2017; Jones et al., 2018; Moyle et al, 2017). Larger studies were focused on robot-based interventions and collected quantitative data only, with two being part of an RCT (Jones et al., 2018; Moyle et al, 2017).

**Table 1.** Characteristics of included studies.

Study number	Author, date	Country published (UK = United Kingdom, USA = United States of America)	Method	Type of intervention	Setting (C = Care home or residential, H = own home)	Sample size (n = )	Method (Q = Questionnaire; OBS = observation; RS = rating scales; I = interviews	Cognitive impairment measure	Outcome measures	Wellbeing focus (S = Social, E = Emotional, P = Physical, PWB = psychological wellbeing)
1	Begum et al., 2015	Netherlands	Qualitative (observation)	Robot-based, assistive robots	H	10	OBS; I	Mini-mental state exam (MMSE)	None	S, E
2	Bemelmans et al., 2015	Netherlands	Quantitative (outcome measures)	Robot-based, pet robots	C	91	RS	None	Effectiveness was measured by a goal attainment scale (IPPA) and coop/Wonca mood scale	S
3	Chu et al., 2017	Australia	Quantitative (observation)	Robot-based, social robots	C	139	OBS	None	Scales were developed from dementia care mapping (DCM) and the well-being/Ill-being scale (WIB)	S, E
4	Cruz-Sandoval and Favela., 2019	Mexico	Qualitative (observation)	Robot-based, social robots	C	12	OBS	None	Video recording to count utterances. Enjoyment was measured using the affect rating scale	S
5	Darragh et al., 2017	New Zealand	Qualitative (interviews)	Robot-based, assistive robots	H	38	I	None	None	S, E, PWB, P
6	D'Cunha, et al., 2021	Australia	Mixed method (pre-test – post-test survey and interviews)	Experience, recreational leisure activity	C	10	OBS, I	None	Environment simulation/apathy - the person–Environment apathy rating (PEAR) scale, behavioural and emotional expression - the engagement of a person with dementia scale (EPWDS), care staff completed the katz index of independence in activities of daily living (KATZ), mood – a five-item visual analogue scale of coloured emoticon faces (mistry et al., 2018).	S, E, P
7	Feng, et al., 2020	Netherlands	Quantitative (observation scales)	Robot-based, social robots	C	16	OBS, RS	None	Engagement - the engagement of a person with dementia scale (EPWDS), emotional - observed emotional rating scale (OERS), apathy related behaviours - people environment apathy rating scales–Apathy subscale (PEAR–Apathy subscale)	S, E
8	Goodall et al., 2021	Norway and Portugal	Qualitative (interviews)	Experience, sensory	C	20	I	None	None	S, E
9	Jones et al., 2018	Australia	RCT quantitative (outcome measures)	Robot-based, pet robots	C	138	RS	Rowland universal dementia assessment scale (RUDAS)	Engagement, mood, and agitation - video observation and cohen mansfield inventory-short form (CMAI-SF).	E
10	Jøranson et al, 2016	Norway	RCT Quantitative (outcome measures)	Robot-based, pet robots	C	27	RS	Clinical dementia rating scale (CDR)	The brief agitation scale (BARs), quality of life in late-stage dementia scale (QUALID)	S, E
11	Laird et al, 2018	UK	Quantitative (outcome measures)	Reminiscence	H	60	Rs, Q	None	The mutuality (positive quality of the relationship between the carer and the care recipient) 15-point scale, the quality carer-patient relationship (QCPR) scale, world health organisation WHO-5 wellbeing scale.	E
12	Lancioni, et al., 2016	Italy	Quantitative (observation)	Reminiscence	H	18	OBS	None	None	E, P
13	Lazar et al, 2016	USA	Mixed method Quantitative (outcome measures) qualitative (interviews)	Experience, recreational leisure activity	C	16	Rs, I	Mini-mental state examination (MMSE).	Self-score quality of life - the quality of life in Alzheimer’s disease (QOL-AD). Depression evaluation - the cornell scale for depression in dementia (CSDD). Total time spent caring in minutes - formal care the resource utilization in dementia. (RUD-FOC)	E, P, PWB
14	Moyle et al, 2017	Australia	RCT Quantitative (outcome measures)	Robot-based, pet robots	C	415	Rs, OBS	None	Engagement, mood, and agitation - **v**ideo observations. Cohen-mansfield agitation inventory short form CMAI-SF)	E
15	Wang et al., 2017	Canada	Qualitative (interviews)	Robot-based, assistive robots	H	10	I	None	None	S, E
16	Wilkerson, et al., 2018	USA	Mixed method (pre-test – post-test survey and interviews)	Experience, social networking	H	12	Q, I	None	Zarit burden interview short form (ZBI-12), the perceived stress Scale-14 (PSS-14), the revised scale for caregiving self- efficacy, and the medical outcomes study (MOS) social support survey.	S, E

There was little consensus in terms of the outcome measures used across the studies, however most studies used rating scales measuring engagement (Cruz-Sandoval and Favela., 2019), mood (Bemelmans et al., 2015), agitation (Jøranson et al., 2016) or all three (D'Cunha, et al., 2021; Feng, et al., 2020; Jones et al., 2018; Moyle et al., 2017). Apathy was measured in two studies (D'Cunha, et al., 2021; Feng, et al., 2020). Quality of life was measured in two studies (Jøranson et al, 2016; Lazar et al, 2016) and wellbeing in two (Chu et al., 2017; Laird et al, 2018). Laird et al (2018) also measured mutuality or the positive quality of the relationship between the carer and the person with dementia. Lazar et al (2016) also measured depression and time spent caring. Wilkerson et al (2018) measured stress and self-efficacy.

Three studies collected post-intervention data through semi-structured interviews with people with dementia and/or family carers (Darragh et al., 2017; Goodall et al., 2021; Wang et al., 2017), and two of these studies were assistive robot interventions. Four studies reported measuring the cognitive impairment of the person with dementia using the Mini-Mental State Exam (MMSE) (Begum et al., 2015; Lazar et al, 2016), the Rowland Universal Dementia Assessment Scale (RUDAS) (Jones et al, 2018), or the Clinical Dementia Rating Scale (CDR) (Jøranson et al., 2016), the remaining studies (n = 12) did not specify this information. The wellbeing emphasis varied between studies, two studies focused on social wellbeing (Bemelmans et al., 2015; Cruz-Sandoval and Favela., 2019), three on emotional wellbeing (Jones et al., 2018; Laird et al, 2018; Moyle et al, 2017), and seven studies on both social and emotional wellbeing (Begum et al., 2015; Chu et al., 2017; Feng, et al., 2020; Goodall et al., 2021; Jøranson et al, 2016; Wang et al., 2017; Wilkerson et al., 2018). One study focused on social, emotional, physical, and psychological wellbeing (Darragh et al., 2017), one on social, emotional, and physical (D'Cunha, et al., 2021), one on emotional and physical (Lancioni, et al., 2016, and one on emotional, physical, and psychological (Lazar et al., 2016).

### Types of interventions

**Figure 2.**
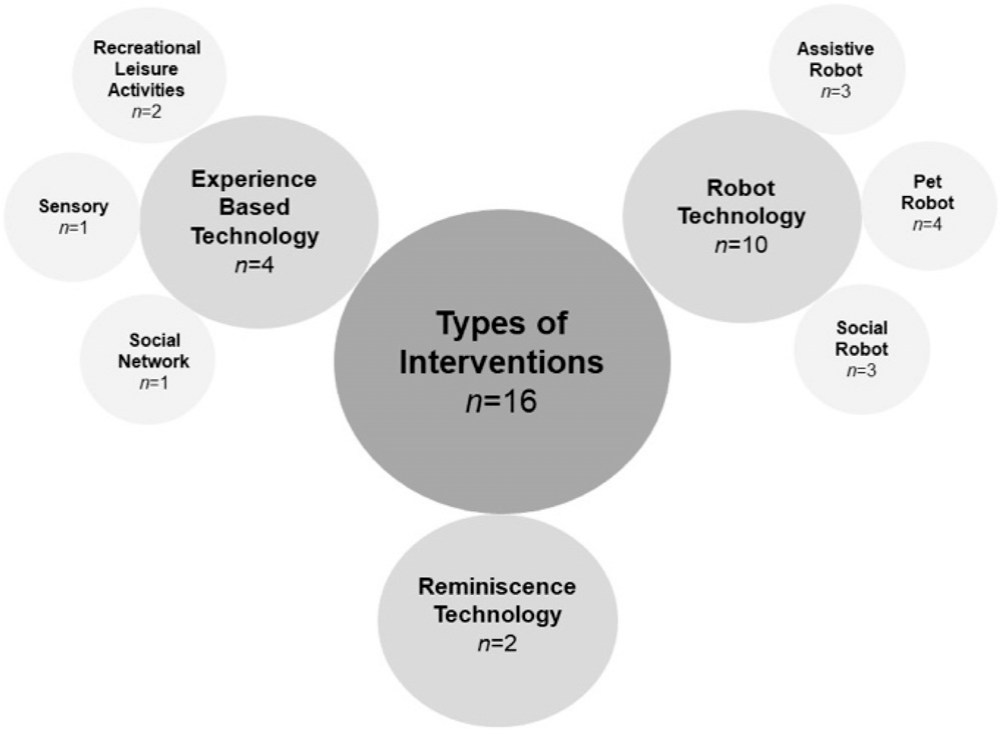
Illustrative map of intervention groups including the robot, reminiscence, and experienced-based technologies.

Studies (n = 16) are grouped according to the type of intervention including robot-based *(n = 10),* experience-based (*n* = 4) and reminiscence (*n* = 2) (Figure 2).

### Robot based interventions

Ten studies reported on robotic technology and have been categorised as three sub-types including assistive *(n* = 3*),* pet *(n* = 4)*,* and social (*n* = 3) robots.

#### Assistive robots

Three studies explored the effects of a collaborative teleoperated human-robot interaction designed to assist older people with dementia with everyday tasks such as making a cup of tea. The teleoperator continuously monitors and initiates conversation throughout the interaction. Darragh et al. (2017) undertook a scoping study and interviewed people with mild cognitive impairment or dementia, their caregivers, and experienced professionals about the design of a homecare robot. They found that a homecare robot could offer both practical and therapeutic benefit in terms of functional support, monitor physical and psychological wellbeing, and provide therapeutic interventions. Suggesting that future research should program homecare robots with scenarios developed from these findings to test feasibility, utility, and acceptability.

Begum et al. (2015) observed a teleoperated robot prompting and asking questions to a person with dementia to complete a tea-making task before guiding them back to their caregiver. This small pilot study highlighted benefits and challenges of using teleoperated robots to support people with dementia in completing everyday tasks. The findings give an indication of what human-robot interventions look like for people with dementia to direct future research efforts. It is suggested research efforts should include a larger study to better understand the needs of the target population and designing an intelligent robot control interface capable of working collaboratively with people with dementia.

Wang et al. (2017) explored the perspectives of people with dementia and their caregivers in semi-structured interviews after using a robot to prompt handwashing and make a cup of tea. People with dementia were open to the idea of robotic assistance yet did not want a robot. Caregivers on the other hand identified opportunities and were more open to robots with several wanting their own robot. Future research should continue conversations between people with dementia, family carers and technology developers to ensure adequate consideration of caregiving relationship factors in robot design.

#### Pet robots

Four studies reported on ‘PARO’ which is an animated robotic baby seal with a swivelling head, legs, and tail. The PARO robot speaks using authentic sounds of a seal, can recognise voices, responds to repeated words, and uses sensors located within the artificial fur (Jøranson et al., 2016). Bemelmans et al. (2015) worked with care professionals to explore the outcomes of therapeutic and caring applications which were developed for PARO to use in a psychogeriatric care setting. After using the PARO, residents did improve their mood scores, and so the study suggests it is a useful intervention if applied in a person-centred way that is tailored to each individual resident with dementia.

Jones et al. (2018) tested PARO to explore whether the severity of cognitive impairment and agitation in older people with dementia predicted the engagement and mood states three time per week over the 10-week intervention program. They found that those with lower levels of cognitive impairment and agitation gained more than those who started with higher levels, recommending that PARO should be restricted to those with low-moderate levels of agitation in clinical practice. Jøranson et al. (2016) evaluated the effects on quality of life when using PARO in a robotic-assisted group activity led by trained nurses with residents living with dementia in a nursing home. They found participants with severe dementia showed stable quality of life and used less psychotropic mediation in the intervention group compared to the control group, recommending that nursing staff could use PARO in group activities to improve quality of life of those with severe dementia.

Moyle et al. (2017) tested the effects of PARO on emotional and behavioural symptoms of dementia when compared to an equivalent soft plush toy in a long-term care facility. They found that participants in the PARO group were more verbally and visually engaged and improved agitation. Although more effective than usual care in improving mood states and agitation, PARO was only more effective than a plush toy in encouraging engagement.

#### Social robots

Three studies highlighted how social robots could benefit people with dementia and their caregivers. Chu et al. (2017) observed how engaging with two baby-faced robots called Sophie and Jack in residential care facilities could improve care quality. The robots were collaboratively designed with Nippon Electric Company (NEC) from Japan to deliver diversion therapy services that involve functions such as face recognition, subject registration and tracking, emotion change recognition, voice vocalization, gestures, emotive expressions, singing, and dancing. The findings show that social robots can improve sensory enrichment, positive social engagement, and entertainment for people with dementia in residential care and that this could have a positive overall impact on quality of life.

Cruz-Sandoval and Favela (2019) designed and observed the effectiveness of a robot called Eva which had incorporated conversational strategies to enable person-robot interactions. Eva could handle simple interactions without human intervention; however, Eva required an operator for more complex communications to send utterances, display emotions, trigger pre-defined activities, and search and play songs. They found that when the conversational strategies were included in the robot people with dementia engaged in more sustained conversations and advocate the need to include appropriate conversational strategies in the design of social robots in the future.

Feng et al. (2020) explored the effects of contextual social interactions between a person with dementia and a robotic sheep. This was building on the researcher’s previous study which created an interactive installation design called LiveNature. The person with dementia used the robotic sheep to interact with a simulated farm environment displayed on a large screen. The findings suggested that the presence of either a static or a proactive robot motivated behavioural engagement, reducing apathy-related behaviours by facilitating purposeful activities.

#### Experience-based

Four studies have been grouped as experience-based technology and have been categorised into three sub-types including recreational leisure activities (*n =* 2), sensory (*n* = 1), and social networking (*n* = 1).

#### Recreational leisure activities

Two studies focused on using digital technology to provide opportunities for recreational leisure activities. D-Cunha et al. (2021) examined the use of a group virtual reality cycling experience for engaging in the physical activity of people with cognitive impairment residing in aged care facilities. Pedal exercisers (Body Charger®GB3030 UBE) were set on the lowest setting and residents cycled along with two pre-recorded videos of local cycle paths which lasted between 20-30 minutes. No differences were observed between conditions for all outcomes except for environmental stimulation. Participants reported the virtual cycling experience to be immersive and challenging and reminisced about cycling earlier in life. The findings of this study support the use of the virtual cycling experience as an immersive and engaging alternative to usual activities, which might encourage higher levels of physical activity in residential aged care facilities.

Lazar et al (2016) used a commercially available system known as iN2LMobile FLEX Lite Package, It’s Never 2 Late, Centennial and CO. The system promotes the physical and mental wellbeing of older adults with memory impairment or dementia who access community-based memory care units. An individual interacts with the touchscreen interfaces to engage in recreational leisure activities. These included social involvement using email, video-calling, and Facebook, entertainment through puzzles, exercise videos, movies, and music, motor involvement with exercise videos, and cognitive training through memory games. They found that staff and family members reported benefits for residents such as enjoyment, interactions and connections with others, and mental stimulation, as well as challenges such as technical and ethical concerns.

### Sensory

One study examined how a multi-sensory experience in care home settings known as SENSE-GARDEN, impacted on how narrative identity and relationships are promoted through individualised and meaningful activities (Goodall et al., 2021). With the support of a trained care worker and family members, older people with dementia interacted with a game designed to improve balance and physical activity, whilst also encouraging reminiscence. Whilst sitting on a stationary bike the individual viewed a video of a known place, old films, a touchscreen device with family photographs, a scent dispensary system that dispensed familiar scents, a large-screen projection of scenic imagery, and surround sound music and soundscapes. They found that the SENSE-GARDEN can stimulate emotional experiences, preserve narrative identity, and foster interpersonal relationships.

The study highlights the complex multitude of factors affecting person-environment interactions in which narrative identity and relationships are constructed. To better understand these factors, future work should adopt a holistic approach to studying new methods of creating meaningful activities in dementia care.

### Social networking

One study examined a 6-week e-health intervention that used social media to promote peer support for caregivers of people with Alzheimer’s disease (Wilkerson et al., 2018). Caregivers interacted with a closed Facebook online support group known as Friendsource by posting and responding to caregiving questions with the aim of developing their personal support networks. They found that caregivers significantly decreased burden (Z = −2.01, *p* < .05) and perceived stress (Z = −2.95, *p* < .01). Emotional and informational support scores were significantly increased (Z = −2.32, *p* < .05). Qualitative data analysis of the intervention identified positive effects in new caregiving knowledge acquisition and application and reduced stress in the acceptance of the caregiving role. Joining social networks in support groups through Friendsource was feasible for caregivers who were familiar with social media and can provide another means of guiding the development of their personal support networks.

### Reminiscence

Two studies have been grouped as reminiscence technology. Laird et al. (2018) used a bespoke I-pad application called InspireD to measure the effect of technology on reminiscence with carer-patient mutuality, quality of relationship, and subjective wellbeing. In their own homes families and the person with dementia engaged in simple reminiscing by using the InspireD application. Following five training sessions each family completed a 3 weekly, 12-week program whilst receiving full telephone IT assistance throughout. They found participants with dementia attained statistically significant increases in mutuality, quality of carer and patient relationship, and subjective well-being (*p*<.001 for all 3) from baseline to endpoint. Carers attained nonsignificant increases in mutuality and quality of carer and patient relationship and a nonsignificant decrease in subjective well-being.

This study suggests that individual-specific reminiscence supported by an iPad app may be efficient in the context of early to moderate dementia. They highlight the need for a robust randomized controlled trial of technology-enabled personalized reminiscence.

Lancioni et al. (2016) examined two technology-aided programs which promoted verbal reminiscence and engagement in mild physical activity for people with moderate Alzheimer’s disease. The verbal reminiscence technology used a computer to display videos and photographs to the participant which they pressed a button to cycle through. Reminders were given when the interaction stopped so the discussion was encouraged throughout. The physical activity technology used a computer playing personalised songs and displaying images to deliver a short simulation which encouraged the individual to participate in arm-raising activities. They found that the participants’ mean percentages of intervals with verbal engagement/reminiscence were below 10 during baseline and control sessions and between above 50 and nearly 80 during the intervention. Also, the mean frequencies of arm-raising responses were about or below four and between about 10 and 19 per session during the baseline and the intervention, respectively. They suggest that further larger studies are needed to investigate the potential of reminiscence and physical exercise.

### Involvement of people with dementia and family carers

Five studies reported using commercially available digital technologies (Bemelmans et al., 2015; Jones et al., 2018; Jøranson et al., 2016; Lazar et al, 2016; Moyle et al., 2017), one of which adapted the technology to the needs of people with dementia (Lazar et al., 2016) (Table 2). A further six studies suggested that they had adapted technology to suit the needs of people with dementia (Chu et al., 2017; Feng, et al., 2020; Goodall et al., 2021; Laird et al., 2018; Wang et al., 2017; Wilkerson, et al., 2018). However, most of the studies did not explain how they had adapted the technology for people with dementia. Only two of these studies included information about how the digital technology being researched was developed (Goodall et al., 2021; Laird et al., 2018). Both studies were based on bespoke digital technology that had previously been developed by the authors and others in user development groups that included people with dementia and their carers.

**Table 2.** Involvement of people with dementia and family carers.

Study	Author, date	Technology adapted for people with dementia	Technology commercially available	Involvement of people with dementia and family carers in design of technology	Researchers observed people with dementia and/or carers using technology	Views of people with dementia and/or carers about the technology collected	Recommend involvement in future research (PwD = Person with dementia, C = Professional carer, F = Family, S = Stakeholder/Designer, N = Not applicable)
1	Begum et al., 2015					X	PwD, C
2	Bemelmans et al., 2015		X		X		C
3	Chu et al., 2017	X			X		PwD
4	Cruz-Sandoval and Favela., 2019				X		N
5	Darragh et al., 2017					X	PwD, C
6	D'Cunha, et al., 2021				X		N
7	Feng, et al., 2020	X			X		N
8	Goodall et al., 2021	X		X		X	N
9	Jones et al., 2018		X		X		N
10	Jøranson et al, 2016		X		X		N
11	Laird et al, 2018	X		X	X		N
12	Lancioni, et al., 2016				X		PwD, C, F
13	Lazar et al, 2016	X	X		X	X	F, C
14	Moyle et al, 2017		X		X		N
15	Wang et al., 2017	X				X	PwD, C, F, S
16	Wilkerson, et al., 2018	X				X	C

Eleven studies collected data about the participants with dementia through outcome measures and observational methods (Bemelmans et al., 2015; Chu et al., 2017; [Bibr bibr13-14713012231178445][Bibr bibr16-14713012231178445][Bibr bibr20-14713012231178445]; Jones et al., 2018; Jøranson et al, 2016; Laird et al., 2018; Lancioni, et al., 2016; Lazar et al., 2016; Moyle et al., 2017). Five studies actively sought the views of people with dementia (Begum et al., 2015; Darragh et al., 2017; Goodall et al., 2021; Lazar et al., 2016; Wang et al., 2017) and one with family carers (Wilkerson, et al., 2018) through qualitative methods (interviews and focus groups). Eight studies suggested a need to involve people with dementia, family carers, and caring and design professionals in future research. One study suggested involving all four groups (Wang et al., 2017), one study referred to people with dementia, family carers and caring professionals (Lancioni, et al., 2016), two studies suggested people with dementia and caring professionals (Begum et al., 2015; Darragh et al., 2017), one study family carers and caring professionals (Lazar et al, 2016), one study people with dementia (Chu et al., 2017), and two studies caring professionals (Bemelmans et al., 2015; Wilkerson, et al., 2018).

## Discussion

This scoping review has examined a range of literature focused on active digital technologies that aim to engage and support the wellbeing of people living with dementia and family carers at home and in care homes. The findings show the potential of digital technologies in enhancing care and support for people with dementia as well as family and professional carers (Goodman-Casanova et al., 2020; Ludden et al., 2019). Although studies appeared to focus on technologies at different levels of technology readiness (European Commission, 2022), many studies were developing ideas or proof of concept; with only five studies evaluating commercially available technologies such as the PARO seal. This had an impact on the methodological approach and the type and amount of data that was collected in each of the studies, making it difficult to evaluate the quality of the included studies. Furthermore, it limited our ability to compare the suitability and sensitivity of existing wellbeing and quality of life outcome measures, particularly as some studies were not yet at the stage of using such measures.

We note a lack of studies that involved people with dementia, family carers, and care professionals in both the design of the intervention and the study itself. Only two studies involved people with dementia in the design of the technology, a finding which concurs with previous studies (Astell et al., 2016; Bejan et al., 2015; Casey et al., 2020; Ke et al., 2020). There were also gaps in terms of studies that sought the views of people with dementia and family carers during data collection, with only six of the studies collecting data directly from people with dementia or family carers. Some of the included studies noted that future active digital technologies should consider the needs and preferences of people with dementia to overcome some of the challenges that occur as people live with the condition, such as changes to communication (Begum et al, 2015; D’Cunha et al, 2021; Joransen et al, 2016; Lazer et al, 2016). For example, in the robot studies, authors noted that as an individual’s dementia progresses, they may find it difficult to communicate effectively with the robot, and this could increase agitation (Bemelmans et al, 2015; Jones et al., 2018; Moyle et al., 2017). It has been suggested that family carers often use technology through ‘bricolage’ or non-conventional ways to address the individual needs of people with dementia (Gibson et al, 2019). Rather than implementing standardised solutions, there is a growing need for real world applications to support people with dementia as they experience changes to memory, speech, mobility, and spatial navigation. Design in the future should focus on how technologies longevity can be improved whilst offering more personalised, adaptive forms of care (Gibson et al., 2019).

In future studies that develop and evaluate active digital technologies for people with dementia, it would be useful to consider the Clarke et al (2020) asset/strengths-based conceptual framework of wellbeing in dementia, as the domains for the measurement of wellbeing reflect equilibrium and potential state of flourishing. Family carers are often relied upon by people with dementia to help to set up and use digital technology, and so are an important group to involve in design too (Begum et al, 2015; Bemelmans et al, 2015; D’Cunha et al, 2021; Goodall et al, 2021; Jones et al, 2018; Lancioni et al; Lazer et al, 2016). We agree that a salutogenesis approach (Simons and Baldwin, 2021), valuing joint interaction between people with dementia and family or professional carers, is key to ensuring that digital technologies capture and support what wellbeing means for people with dementia, as they live with the condition. Designing personalised digital technology that can be used by the person with dementia themselves, and/or with family carers and professional carers is key to ensuring widest adoption (Begum et al, 2015; Bemelmans et al, 2015; Chu et al, 2017; Darragh et al, 2017; Jones et al, 2018; Lazer et al, 2016; Wilkerson et al, 2018). In our review the experience-based technology appeared to capture the essence of personalisation for people with dementia, and by being able to identify individual interests the technology also supported relationship building with family carers (D’Cunha et al, 2021; Goodall et al, 2021; Lazer et al, 2016; Wilkerson et al, 2018)

## Strengths and limitations

Our review includes 16 studies focused on people with dementia at home and in care homes, with the intention of building on existing reviews that address how digital technologies support meaningful engagement (Luscombe et al, 2021; Neal et al., 2020). The focus in this review has been on active digital technologies for people with dementia at home and in care homes, and studies appear to have focused in one or other setting rather than in both. To support a continuum of care, we suggest a need for digital technologies that are adaptable in any residential setting including own homes, care homes and hospitals. There also remains wider questions that we have been unable to answer in this review:

- Will this technology enable people with dementia to remain in their own homes for longer (reduce isolation/loneliness) rather than go into care homes?
- Is there a link between introducing this technology early at home and then being able to continue using it in care homes (for the person themselves and family carers)?
- What is the added value of digital technology in enhancing wellbeing for people with dementia, as family and/or staff often busy and not able to interact as much as they would like?
- How does the technology account for the changes in cognitive, emotional, and physical deficits of people with dementia and how can this be captured in the context of wellbeing and useability?

Our findings are useful in identifying areas for further research but are limited by the methodological quality of the included studies, with many small studies reporting on one quantitative or qualitative approach/method involving a purposive or small random sample (such as Cruz-Sandoval and Favela, 2019; Feng et al, 2020; Goodall et al, 2021; Joransen et al, 2016).

## Future research directions

Much of the active digital technologies we looked at in this review were designed for people with mild to moderate dementia rather than severe dementia. There is a need for future research to develop and evaluate innovative solutions that have impact across the spectrum of the dementia journey, particularly if the aim is to use in social care settings (Darragh et al, 2017; Jones et al, 2018; Laird et al, 2018). As some of the included studies suggest, the best way to achieve this is through multidisciplinary teams, including people living with dementia and family carers, people working in care, social scientists such as sociologists and psychologists, and digital developers. We advocate that using coproduction methodologies will enable equity of voices throughout the development and evaluation phases, ensuring that digital solutions are fit for everyone’s purpose. Future research therefore should develop the evidence base with coproduced digital technologies that address the social, emotional, physical, and psychological wellbeing of people with dementia and are evaluated using robust methodologies. Future research could also examine digital exclusionary factors as have been highlighted by National Voices (2021), and whether there is scope for a ‘digital toolkit for all’ to improve availability, accessibility and promote inclusion.

## Supplemental Material

Supplemental Material - Effectiveness of digital technologies to engage and support the wellbeing of people with dementia and family carers at home and in care homes: A scoping review Click here for additional data file. Supplemental Material for Effectiveness of digital technologies to engage and support the wellbeing of people with dementia and family carers at home and in care homes: A scoping review by Lyndsey Bradley, Shanti Shanker, Jane Murphy, Lee-Ann Fenge, and Michelle Heward in Dementia
