# Supplementary material for: Effectiveness of digital technologies to engage and support the wellbeing of people with dementia and family carers at home and in care homes: A scoping review
Source: Dementia (London). 2023 May 26;22(6):1292–313. doi: 10.1177/14713012231178445 (PMC10336719; doi:10.1177/14713012231178445)
Supplement: Supplemental Material - Effectiveness of digital technologies to engage and support the wellbeing of people with dementia and family carers at home and in care homes: A scoping review [file sj-pdf-1-dem-10.1177_14713012231178445.pdf]

Supplementary material: Example of database search terms and results

**Medline database search on 17/06/2021**		
MH = mesh and exploded to include medical terms and sub-categories<br>S = Search		
**Search Number**	**Search term**	**Number of results**
S3	(MH "Digital Technology") OR (MH "Technology+") or digital technology OR (MH "Digital Divide")	**451,440**
S4	(MH "Dementia+") OR (MH "Alzheimer Disease") or alzheimer* or dementia	**285,693**
S6	((MH "Dementia+") OR (MH "Alzheimer Disease") or alzheimer* or dementia) AND (care* or patient or person or health professional)	**133,132**
S7	((MH "Dementia+") OR (MH "Alzheimer Disease") or alzheimer* or dementia) AND (hosp* or care* or community or home)	**102,415**
S10	S3 and S4	**1802**
S29	S3 and S4 and S6 and S7 and S10	**436**
S30	S29 **included full text and dates 2015 to 2021**	**251**
	**After studies removed for not meeting inclusion criteria**	**141**
	**After duplicates removed in EndNote**	**102**
	**After further removal of studies not meeting criteria on closer review**	**86**
	**Further checks by first author**	**79**
